# Supplementary figures and images for: Coxsackievirus Group B3 Has Oncolytic Activity against Colon Cancer through Gasdermin E-Mediated Pyroptosis
Source: Cancers (Basel). 2022 Dec 15;14(24):6206. doi: 10.3390/cancers14246206 (PMC9776948; doi:10.3390/cancers14246206)

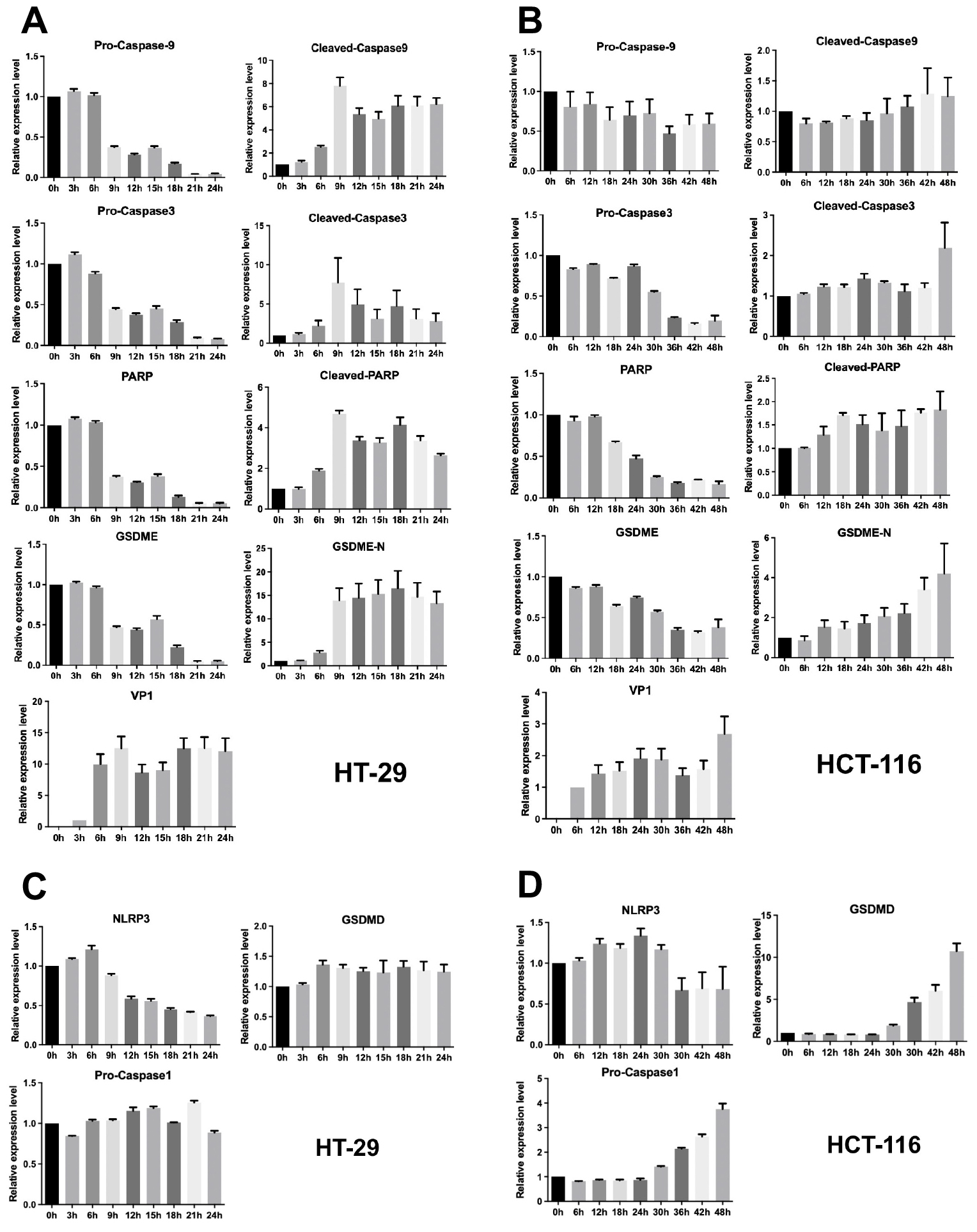

Supplement: Supplementary file 1 [file cancers-14-06206-s001.zip › Figure S1.jpg]

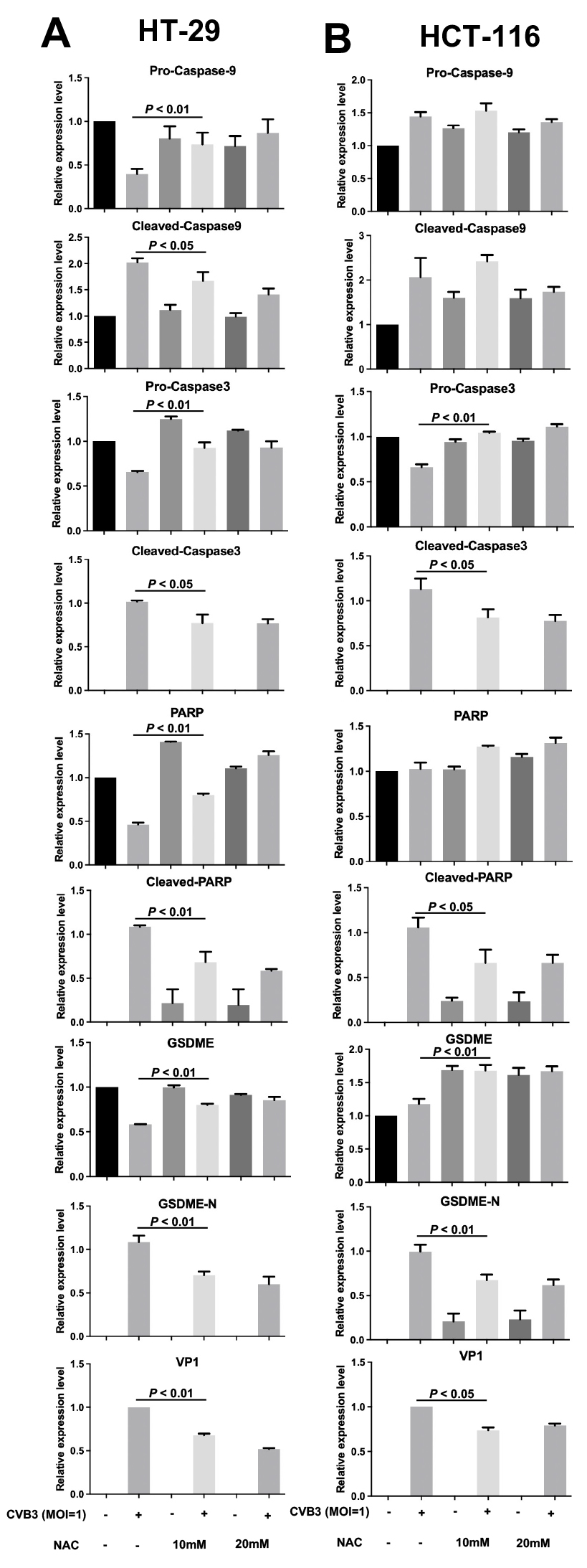

Supplement: Supplementary file 1 [file cancers-14-06206-s001.zip › Figure S2.jpg]

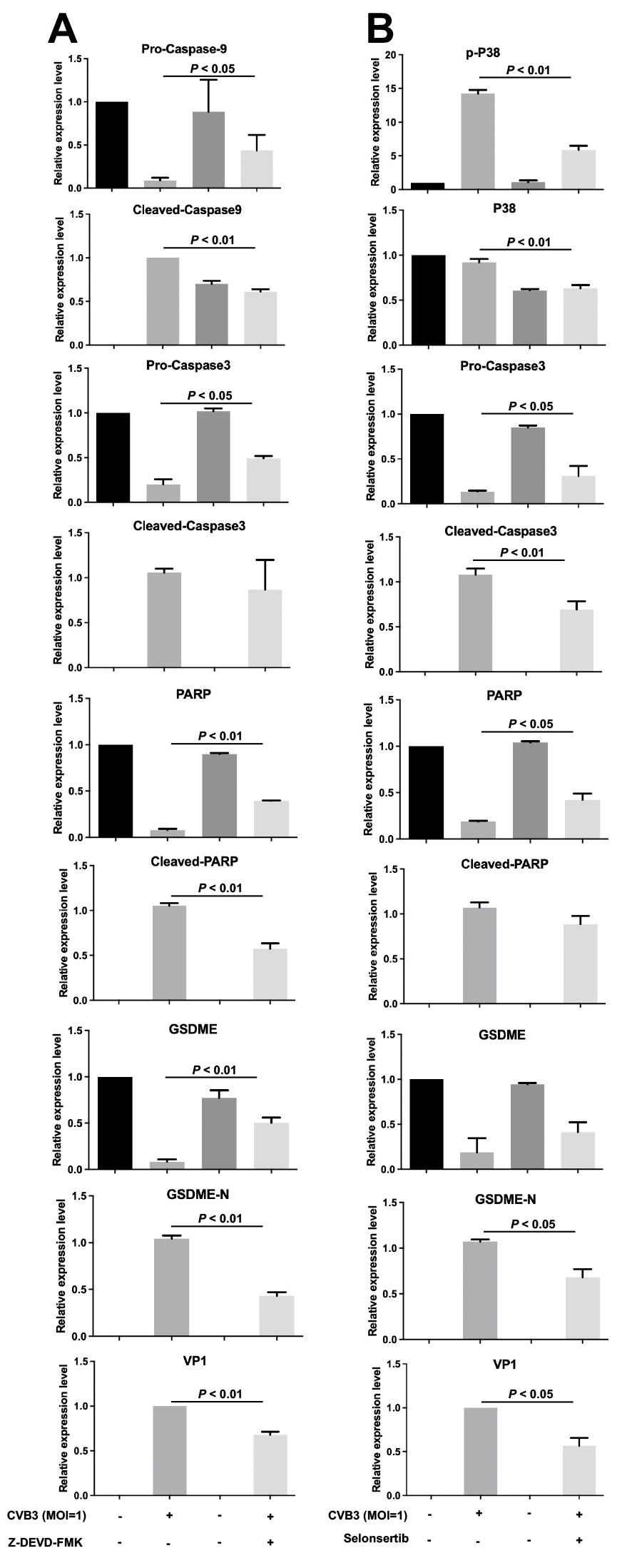

Supplement: Supplementary file 1 [file cancers-14-06206-s001.zip › Figure S3.jpg]

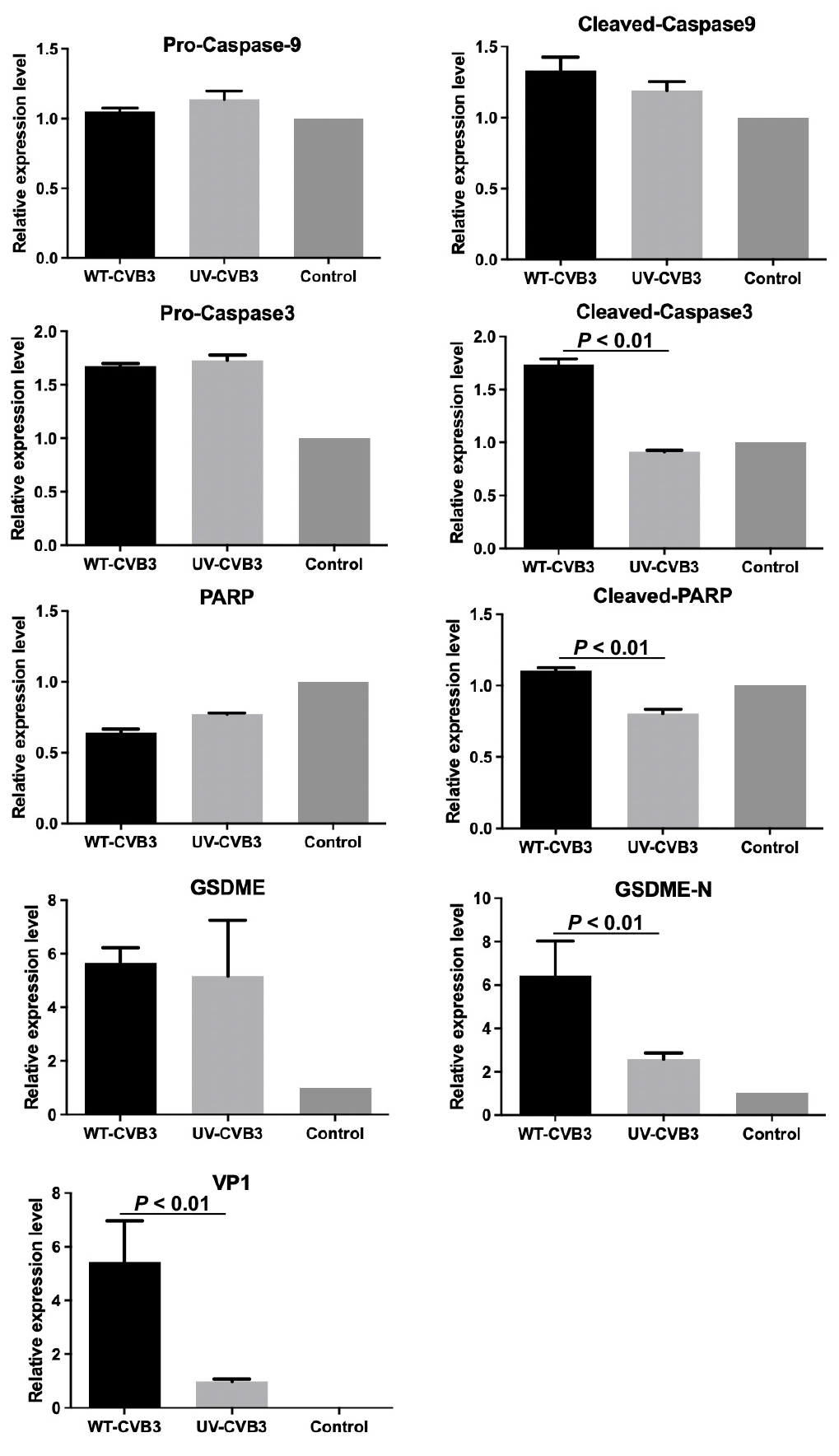

Supplement: Supplementary file 1 [file cancers-14-06206-s001.zip › Figure S4.jpg]
